# Hydrophobic Ion Pairing of Polymyxin B with Oleic Acid: A Dissipative Particle Dynamics Simulation Study

**DOI:** 10.3390/pharmaceutics17050574

**Published:** 2025-04-27

**Authors:** Nargess Mehdipour, Sima Kiani, Hossein Eslami

**Affiliations:** Department of Chemistry, College of Sciences, Persian Gulf University, Boushehr 75168, Iran; mkiani1993@gmail.com (S.K.); h.eslami@theo.chemie.tu-darmstadt.de (H.E.)

**Keywords:** dissipative particle dynamics, drug delivery, drug release, hydrophobic ion pairing, polymyxin B, nanoclusters

## Abstract

**Background:** Hydrophobic ion pairing is a technique for reducing the hydrophilicity of charged molecules (drugs) by pairing them with oppositely charged hydrophobic counterions. This method is used to control the solubility of charged molecules in a solvent and is of particular importance in drug delivery. **Methods:** Dissipative particle dynamics simulations were performed to provide a microscopic understanding of hydrophobic ion pairing in polymyxin B (PMB) and oleate (OA) ions. Solvents and ions were explicitly included in the simulations. **Results:** We investigated the effects of relative concentrations of PMB and OA (the charge ratio), solvent philicity, and the concentrations of PMB and OA at a fixed composition on the structural stability and the hydrophobicity of the ion paired cluster, as well as the kinetics of assembly. The maximum hydrophobicity belongs to PMB:OA charge ratio 1:1. The clustering efficiency in mixed ethanol–water solutions decreases with the increasing ethanol content of water. The dynamics of PMB/OA exchange between hydrophobic cluster and the surrounding solution reveal two distinct relaxation processes, whose relaxation times differ by two orders of magnitude. **Conclusions:** The hydrophobicity of the cluster is controlled by the charge ratio. The core of the ion paired cluster acts as the primary barrier and its surface layer acts as the secondary barrier against alcohol permeation into it. The exchange of surface PMB/OA ions with the surrounding is a much faster dynamic process than the establishment of equilibrium between the PMB/OA ions in the cluster and the solution. The time scale for the slower process provides useful information on the rate of drug release from the hydrophobic ion paired complex.

## 1. Introduction

The term hydrophobic ion pairing refers to a process in which the charged groups on a hydrophilic ion and those on a hydrophobic counterion form hydrophobic ion pairs. Replacement of the polar counterions with the hydrophobic species and covering the hydrophilic groups with the hydrophobic groups increase the hydrophobicity of the ion pair complex. This process is used to control solubility of charged molecules in a solvent, which is of particular importance in such applications as enhanced protein stability [1], increased bioavailability of drugs [2], colloid stability [3], and increased hydrophobicity of peptides for their loading in lipidic self-emulsifying drug delivery systems [4,5].

In drug delivery systems, factors such as the drug solubility and desorption of surface adsorbed drug control the drug release rate. The conventional methods for tuning the drug release rate in such devices include surface modifications of the nanoparticles, through changing their composition, and encapsulation of prodrugs [6]. Both methods have their own disadvantages, which makes the delivery system more complex. However, by properly choosing counterions of various degrees of hydrophobicity, the drug release rate can be controlled through formation of hydrophobic ion pair complexes between the drug and the hydrophobic counterions. In addition, the hydrophobic ion pair complex has a higher solubility in nonpolar solvents, which in turn leads to increased drug loading [6]. Over the past decades, hydrophobic ion pairing has been the focus of active research into increasing the lipophilicity of drugs for their incorporation into hydrophobic nanocarriers [7,8,9].

Of particular importance is the application of this technique for oral drug delivery. It is worth mentioning that, in majority of cases, increased water solubility of the drug has been the target of research. Decreased water solubility of drugs has, however, been less extensively studied. Recently, hydrophobic ion pairing has been employed to precipitate small molecules [10]. Highly soluble drugs, such as polymyxin B (PMB), a hydrophilic peptide containing five positively charged lysine groups, are however, less well studied. A hydrophobic counterion such as the oleate ion (OA) highly increases the lipophilicity of PMB [11]. Polymyxin B is a drug used for treatment of infections of the urinary tract, meninges, and blood stream, caused by susceptible strains of *Pseudomonas aeruginosa* [12]. Upon drug administration, the drug mostly adheres to pulmonary mucus and the infection sites are not efficiently exposed to it [11]. Hydrophobic ion pairing of the drug with hydrophobic counterions, to decrease water solubility of the drug, is a useful technique in such a situation.

As well as experimental investigations, as discussed, molecular simulations are useful tools for providing a molecular level understanding of cluster formation [13], the stabilities of ion paired clusters, and the kinetics of ion pair formation [14]. Computer simulations have been used to investigate ion pairing, utilizing implicit or explicit solvent models [15,16,17,18]. Because the specific interactions between ions and hydrophilic/hydrophobic sites with solvents play a major role in stabilizing hydrophobic ion paired complexes, explicit solvent models are preferable over implicit solvent models [19]. However, in these models, a vast amount of computational resources are used to carry out the simulation of solvent molecules [20]. Therefore, a coarse-grained representation [21,22] of the drug, the counterion, and the solvent are invoked to reduce the computational cost. This allows one to simulate much larger size systems over longer time scales, which are not achievable in atomistic simulations [21,22].

Compared to atomistic simulations, in coarse-grained simulations, faster degrees of freedom are eliminated by lumping a group of atoms into a coarse-grained bead. This reduction in the number of degrees of freedom allows access to longer time- and length-scales than would be achievable in atomistic simulations [23]. Nucleation and clustering of hydrophobic ion pairs occur over a wide range of scales, from single molecule, i.e., nanoscale, to clusters composed of many ion pairs, i.e., mesoscale. Therefore, a mesoscale simulation scheme is necessary to extend simulation length and time-scales to scales appropriate for formation of hydrophobic ion paired clusters. For this purpose, we employ a mesoscale simulation scheme, namely, the dissipative particle dynamics (DPD) [24,25] simulation method. We construct DPD models of PMB cation, OA anion, solvent (water and ethanol), and ions (Na^+^ and Cl^−^) to investigate hydrophobic ion pairing of PMB with OA in the presence of solvent (water). We study the influence of solvent philicity, charge ratio, concentration of PMB and OA ions on the formation of ion paired clusters, and the dynamics of ion paired clusters.

## 2. Materials and Methods

### 2.1. Method

DPD is a particle-based technique, designed for simulation of soft matter systems. In this coarse-grained approach a group of atoms constitute a DPD bead, and the motion of beads is governed by Newton’s law. The DPD beads are subject to three types (conservative, dissipative, and random) of pair-wise forces [24,25]. The random force, $F^{R}$, is added to the conservative force, $F^{C}$, to take into account the lost degrees of freedom, and the dissipative force, $F^{D}$, is responsible for the viscous effects and acts on two DPD beads to reduce their relative velocity. The conservative, dissipative, and random forces between two DPD beads *i* and *j* read as:

(1) $$F_{ij}^{C}=A_{ij}\left(1-\frac{r_{ij}}{r_{C}}\right){\hat{r}}_{ij}$$

(2) $$F_{ij}^{D}=\gamma \left(1-\frac{r_{ij}}{r_{C}}\right)\left({\hat{r}}_{ij}\cdot v_{ij}\right){\hat{r}}_{ij}$$

and

(3) $$F_{ij}^{R}={\left(1-\frac{r_{ij}}{r_{C}}\right)}^{1/2}\theta_{ij}∆t^{-1/2}{\hat{r}}_{ij}$$

where $r_{ij}$ is the distance between particles *i* and *j*, $A_{ij}$ is the DPD repulsion parameter, ${\hat{r}}_{ij}$ *=* $r_{ij}$*/*$r_{ij}$*,*γ is the dissipation strength, $v_{ij}$ is the relative velocity of ij pair, $\theta_{ij}$ is a random variable with a Gaussian distribution and unit variance, δ is the noise strength, and ∆t is the time step. All three forces are short range and have a range equal to the cutoff distance, $r_{C}$.

While the random force heats the system up, by increasing the bead velocities, the dissipative force cools it down. Espanol and Warren [26] showed that the constant temperature–constant volume ensemble is recovered provided that the following balance condition between the dissipation and random strengths exists:

(4) $$\delta ={\left(2\gamma k_{B}T\right)}^{1/2}$$

where $k_{B}$ is the Boltzmann constant and *T* is the temperature. Therefore, the dissipative and random forces act together as the DPD thermostat. While the conservative force is responsible for the thermodynamic behavior of the system, the dissipative and random forces take into account its hydrodynamic behavior. The cutoff distance, $r_{C}$, bead mass, *m*, and the thermal energy per bead, $k_{B}T$, serve as reducing units for length, mass, and energy, respectively.

In this work, we have constructed a DPD model, based on the four-to-one mapping scheme of the MARTINI model [27,28], as is conventionally performed for varieties of soft matter systems [29,30]. In the following sections, we present the DPD mapping scheme and the DPD parametrization for simulation of hydrophobic ion pairing in PMB and OA, in the presence of solvent and charges.

### 2.2. Model and Simulation Details

**Figure 1:**
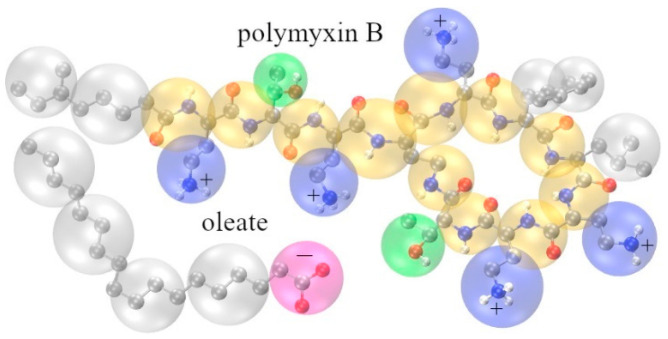
Representation of the mapping scheme adopted for polymyxin B and oleate ions. Color codes in atomistic model: N (blue), O (red), and C (gray). Color code for coarse-grained (DPD) model: CH_2_CH_2_NH_3_^+^ (blue), CONHCH_2_ (orange), CH_3_CHOH (green), CHCHCH and/or CH_2_CH_2_CH_2_CH_2_ (gray), CH_2_COO^−^ (magenta).

The four-to-one mapping scheme of the Martini model [27,28] is used to represent the DPD interaction centers (beads) for the PMB cation and OA anion, as shown in Figure 1. For the solvent, four water molecules, which have a total volume of 0.12 nm^3^, correspond to a single DPD bead. At a reduced density ρ = 3, a cube of volume $r_{C}^{3}$ contains 3 water beads. Therefore, the cutoff distance $r_{C}$ = 0.711 nm. The molar density of ethanol at 298 K is 787.5 kg/m^3^ [31], i.e., the volume of a single ethanol molecule is 0.097 nm^3^. In other words, a single ethanol DPD bead contains 1.24 molecules. Based on the mapping scheme shown in Figure 1, there are five types of DPD beads for PMB and OA: positively charged CH_2_2212CH_2_–NH_3_^+^ beads, negatively charged CH_2_–COO^−^ beads, polar groups with hydrogen bond donor-acceptor capacity (NH–CO–CH), nonpolar groups with hydrogen bond donor capacity (CH_3_–CH–OH), and apolar C_3_H_6_ or C_4_H_9_ groups. In addition, there are solvent beads (H_2_O)_4_, hydrated counterion beads, Na^+^(H_2_O)_4_, Cl^−^(H_2_O)_3_, and ethanol beads, (C_2_H_5_OH)_1.24_.

To reproduce the compressibility of water at room temperature, the DPD repulsion parameter is set to 100 for water–water interactions (*A_ww_* = 100) [32]. Similarly, for beads of the same type, the repulsion parameter is set to 100 (the $A_{ij}$ for the interaction of charged beads with CH_3_CHOH beads is set to 102.5). All other repulsion parameters for dissimilar beads are taken from the literature [32,33]. For charged beads, the charges are explicitly taken into account; the electrostatic interactions are calculated based on a method in which the charges are distributed over DPD beads in the form of Gaussians of tunable widths [34]. The bonded beads are connected through a harmonic potential:

(5) $$U_{b}=\frac{1}{2}k_{b}{\left(r-r_{0}\right)}^{2}$$

where $k_{b}$ is the spring constant and $r_{0}$ is the equilibrium bond length. The bond bending stiffness is described according to the following potential:

(6) $$U_{\theta }=k_{\theta }\left(1-\cos\left(\theta -\theta_{0}\right)\right)$$

where $k_{\theta }$ is the force constant and $\theta_{0}$ is the equilibrium angle between the two connected bonds. All the DPD interaction parameters are reported in Table 1.

(b) parameters for bonds and angles:

The equilibrium bond lengths $r_{0}$ = 0.47 for CH_2_CH_2_NH_3_^+^–NHCOCH and CH_2_COO–C_4_H_8_ bonds. For the rest of the bonds $r_{0}$ = 0.59. For all bonds the spring constant $k_{b}$ = 512. All equilibrium bond angles in polymyxin B are 180°, except the side chain angles which are 120°. For the oleate anion, all angles are equal to 180°, except the angle whose vertex bead encompasses the double bond, which is 120°. For all angles, the force constant $k_{\theta }$ = 6.

All simulations are run using the simulation package YASP [35]. The equations of motion are integrated using the velocity–Verlet integration scheme with a time step 0.024. As the natural DPD unit of time $\tau =r_{C}{\left(m/k_{B}T\right)}^{0.5}$, the time step in real units is 0.09 ps. The DPD simulations are run for at least 10^6^ (corresponding to 3.75 μs). The first 5 × 10^5^ is considered as the equilibration cycle, monitored as the stage at which the concentration of PMB or OA ions in the solution become constant, and the rest is used for data collection. The aggregates are distinguished from PMB and OA ions based on a distance criterion, i.e., individual ions are regarded as being involved in the same aggregate if their centers-of-mass locate within a distance 0.75$r_{C}$ from each other.

We simulated hydrophobic ion pairing in the PMB cation and OA anion at a constant temperature (300 K) and constant volume. Because, upon cluster formation, the concentrations of free PMB and OA ions in the solution decreases, simulation of small size systems is envisaged with a significant size effect [36,37]. Another important issue in simulations of this type is that cluster formation is a stochastic event [38]. In other words, a single simulation does not provide statistically significant results of the clustering process. Therefore, many simulations on large size systems are needed to provide statistically significant results of hydrophobic ion pairing. On this basis, we performed DPD simulations on many samples, over a wide range of concentrations of PMB and OA in water and/or water–ethanol mixtures. The compositions of samples and the dimensions of the cubic simulation box are shown in Table 2. In addition, simulations were performed on large size systems (up to 1,238,400 DPD beads) in large simulation boxes (box length ≈ 74$r_{C}$). Initial configurations of each sample are prepared by randomly distributing cations and anions in a cubic box.

## 3. Results and Discussion

### 3.1. Cluster Formation in Polymyxin B and Oleate

Ion pair formation between the PMB cation and OA anion is studied at a constant temperature (300 K) and constant volume. Because the tendency for cluster formation depends on the concentrations of both species, we chose a wide range of concentrations of both species in water and water–ethanol solvents. The charge ratio is an important factor, which determines the morphology and the hydrophobicity of the cluster (see Section 3.4). Previous reports [39] indicate that at a charge ratio 1:1 (one PMB cation per 5 OA anions), bigger spherical clusters are formed. Therefore, we report the results of this section for clusters formed at a charge ratio 1:1.

**Figure 2:**
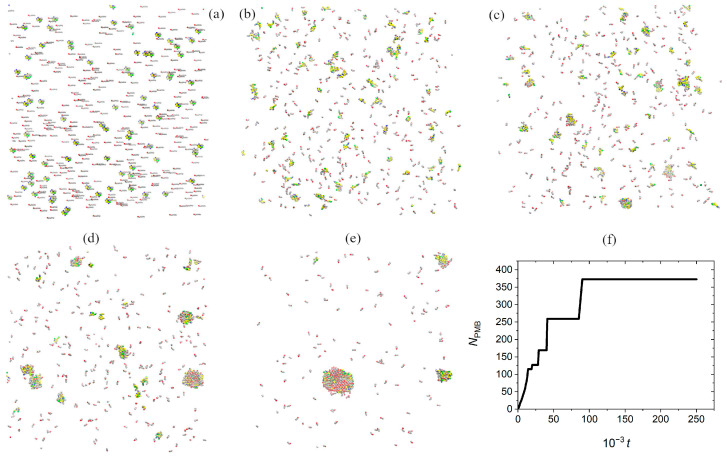
(**a**–**e**) Snapshots of the simulation box, representing hydrophobic assembly in polymyxin B with oleate in a system containing 80 PMB cations at the PMB:OA charge ratio 1:1. Color coding is the same as Figure 1. (**f**) Time dependence of the largest cluster size, defined in terms of number of polymyxin B cations in the cluster, in a system containing 400 PMB at the PMB:OA charge ratio 1:1.

Figure 2 shows snapshots of the simulation box, characterizing cluster formation at different stages of assembly. Starting from a random configuration of PMB and OA in water (Figure 2a), during the course of DPD simulations, the cations and anions diffuse through the solution. The oppositely charged PMB and OA ions can attract each other at short distances to form small size initial precursors (Figure 2b). Such small aggregates are not stable; they associate and dissociate due to competition between Coulombic attractions and thermal fluctuations. However, when the size of aggregates exceeds a threshold value, they are stable enough not to dissociate back to the initially isolated ions. Typical stable clusters can be seen in Figure 2c. The smaller clusters grow by successive accumulation of oppositely charged ions, due to Coulombic interactions. Over this regime, the cluster size varies smoothly with time (Figure 2f). In other words, multiple stable moderate size clusters form in the simulation box. Formation of many small clusters in the system is an entropically favorable process [40]; however, formation of large clusters is favorable from the point of view of the interfacial free energy [41]. Following the initial (smooth) cluster growth regime, the cluster growth rate stops until diffusion brings the clusters together. Attachment of stable clusters causes a sudden increase in the cluster size (Figure 2d,e). With the passage of time, all smaller clusters merge into a single large (stable) cluster (Figure 2e). Such a step-growth mechanism of hydrophobic ion pairing is seen in Figure 2f. A similar step-growth mechanism has been reported for crystallization of colloidal particles [42].

**Figure 3:**
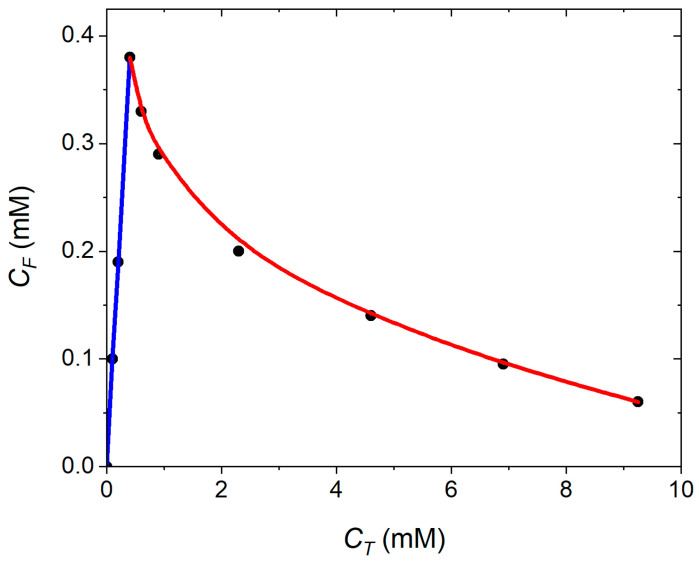
Dependence of concentration of free polymyxin B, $C_{F}$, on its total concentration, $C_{T}$, in the solution at PMB:OA charge ratio 1:1. The markers represent simulation results, and the blue line is the best fit through simulation results over the linear regime. The red curve shows the best fit through calculated results according to Equation (7).

To provide insight into the precipitation conditions, we Figure 3 shows the concentration of free (*N* ≤ 3 PMB ions), $C_{F}$, versus the total concentration of PMB, $C_{T}$, in the solution. At low PMB concentrations, $C_{F}$ = $C_{T}$, hence, no cluster forms. The highest concentration at which $C_{F}$ = $C_{T}$ corresponds to the onset of clustering. At higher PMB concentrations, the turnover in the $C_{F}$ vs. $C_{T}$ indicates that a fraction of PMB ions form ion paired clusters with OA ions. Due to Coulombic attractions between the PMB and OA ions, an increase in the total PMB concentration causes a decrease in the concentration of free PMB ions in the solution. This trend is in agreement with experimental reports on ion pairing between PMB and OA ions and with previous simulation and experimental reports on micellization in ionic surfactants [43,44].

Following the literature on the micellization of ionic surfactants in water [44,45], the empirical relation by Sanders et al. [46] was employed to find the critical concentration, $C_{C}$, of PMB (at the PMB:OA charge ratio 1:1), which leads to ion pair formation. The Sanders et al. equation [46] reads as:

(7) $$\log\left(C_{F}\right)=\left(1+\beta \right)\log\left(C_{C}\right)-\beta \log\left(\frac{\left(1-\beta \right)\left(C_{T}-C_{F}\right)+C_{F}}{1-\bar{V}C_{T}}\right)$$

where *β* is the degree of association of counterions with the ion pair forming species and $\bar{V}$ is the molar volume of ion paired cluster (assumed to correspond to a density of 1000 kg/m^3^). The calculated $C_{F}$ vs. $C_{T}$ points in Figure 3 were fitted according to Equation (7), with both *β* and the critical concentration of PMB needed for ion pair formation with OA as the fitting parameters. The fitted values of *β* = 0.81 and $C_{C}$(PMB) = 3.8 mM.

### 3.2. Structure of Ion Paired Clusters

**Figure 4:**
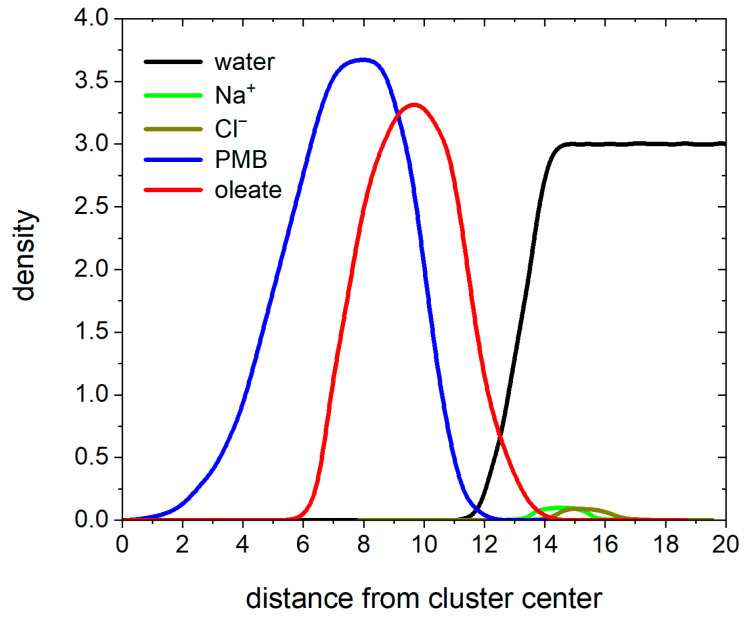
Density profiles for PMB, OA, counterions, and water as a function of distance from the cluster center-of-mass for a system containing 800 PMB and OA at a charge ratio 1:1.

We characterized the structure of hydrophobic ion paired clusters in terms of distributions of PMB and OA ions as well as solvent and counterions in the cluster. Assuming that the cluster has a nearly spherical shape, we show in Figure 4 the calculated density distributions as a function of distance from the center-of-mass of cluster. The PMB cation is mostly concentrated in the interior core of the cluster and extending to the surface of the cluster. On the other hand, the OA anion mostly covers the exterior part of the cluster; it makes the cluster hydrophobic. The counterions (Na^+^ and Cl^−^) are located at the surface of the cluster. The outermost part of the cluster is composed of both hydrophilic and hydrophobic groups; however, a larger fraction of surface is covered with the hydrophobic groups. Water is mainly solvating the charged surface beads; it is excluded from the core, but it penetrates to the surface of cluster.

**Figure 5:**
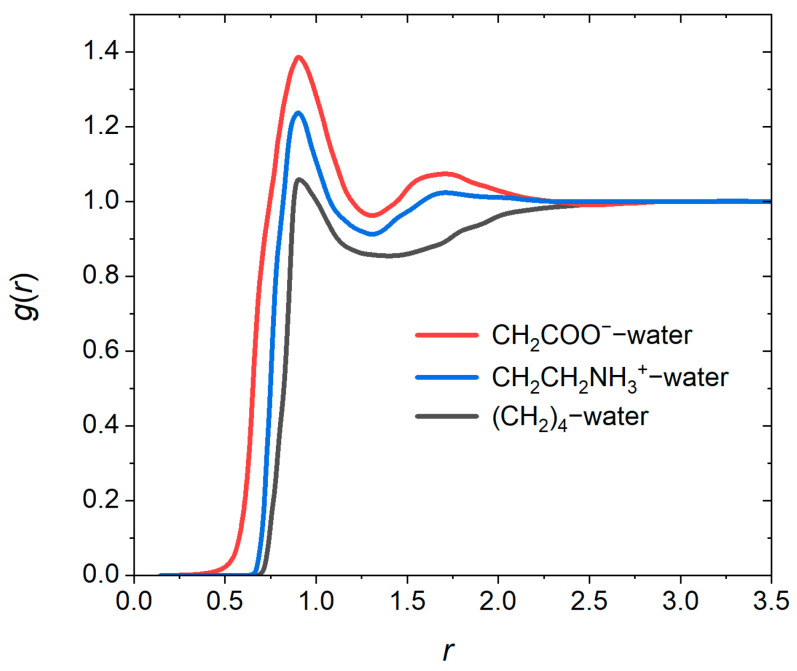
Radial distribution functions for water–CH_2_CH_2_NH_3_^+^, water–CH_2_COO^−^, and water–C_4_H_8_ pairs. The distributions are calculated for pairs locating inside a cone of opening angle 90°, whose vertex is centered on the considered bead on the surface of cluster and orients along a line connecting the center-of-mass of cluster to the vertex.

To examine the structure of the cluster at the water interface, we have also shown, in Figure 5, the radial distribution functions, *g*(*r*), for water–CH_2_CH_2_NH_3_^+^ beads of PMB, water–CH_2_COO^−^ beads of OA, and water–hydrocarbon tail of OA. Because in this case part of the space is occupied by the cluster itself, we have calculated the *g*(*r*) curves for pairs falling into a cone of opening angle 90°. The vertex of the cone is located on the bead of interest on the cluster surface and it opens away from the cluster surface, along a line connecting the center-of-mass of cluster to the cone vertex. The *g*(*r*) peak for water–CH_2_COO^−^ is indicative of a strong correlation between the charged OA groups in the cluster and water. Because some PMB ions extend to the surface of the cluster, a less strong *g*(*r*) peak for water–CH_2_CH_2_NH_3_^+^, than that of water–CH_2_COO^−^, is observed. The *g*(*r*) curve for water–CH_2_COO^−^ starts at shorter distances and shows stronger correlations than that of water–CH_2_CH_2_NH_3_^+^. In addition, the tail C_4_H_8_ and/or C_4_H_9_ beads of the OA are also in contact with water. This indicates that OA partially forms a hydrophobic layer at the surface of the cluster. The interface thickness for distributions of water at the surface of the cluster is ≈2.7$r_{C}$ (corresponding to ≈2 nm). This thickness agrees with previous calculations [39] and experimental X-ray scattering data [47] for micellar aggregates.

### 3.3. Effect of Solvent Philicity

**Figure 6:**
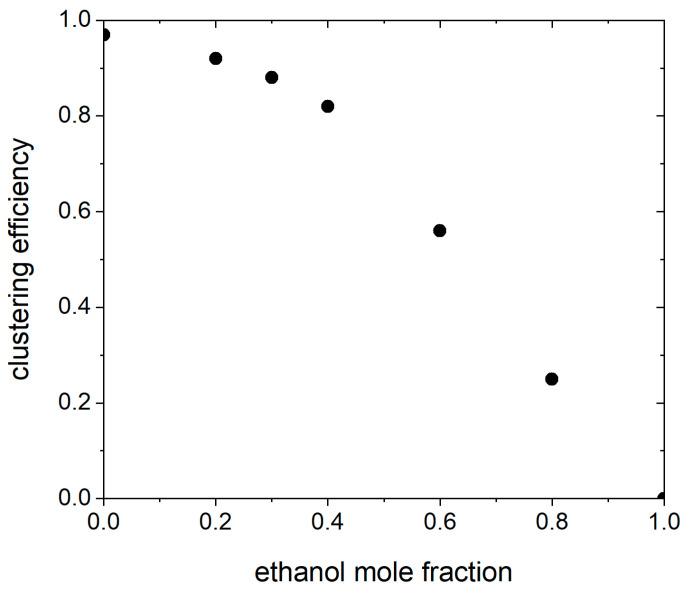
Efficiency of ion pair clustering between polymyxin B and oleate in water–ethanol mixtures, in a system consisting of 200 PMB and OA at the charge ratio 1:1.

To investigate the influence of solvent philicity on hydrophobic ion pairing, we examined the tendency for ion pair formation in water–ethanol mixtures, over the whole range of compositions. This is compatible with experimental investigations, where the hydrophobic ion pair is solubilized in a solvent containing an organic phase, to form subsequent nanocarriers for advanced drug delivery [48]. The calculated clustering efficiencies [39] as a function of ethanol mole fraction are shown in Figure 6. According to the literature [39], we defined the clustering efficiency as the fraction of time a single cluster exists in the simulation box. Increasing the mole fraction of ethanol up to a threshold value (0.4) has no notable effect on the hydrophobic ion pair clustering efficiency. A further increase in the ethanol mole fraction causes a quick decay in complexation efficiency. In fact, the hydrocarbon tail of the OA dissolves better in the more hydrophobic solvent (ethanol) than in water; hence, increasing the ethanol content of water disintegrates the cluster. These findings are in agreement with experimental findings and with previous atomistic simulation results for hydrophobic ion pairing of OMB with OA in water–methanol mixtures [39].

### 3.4. Effect of Charge Ratio

**Figure 7:**
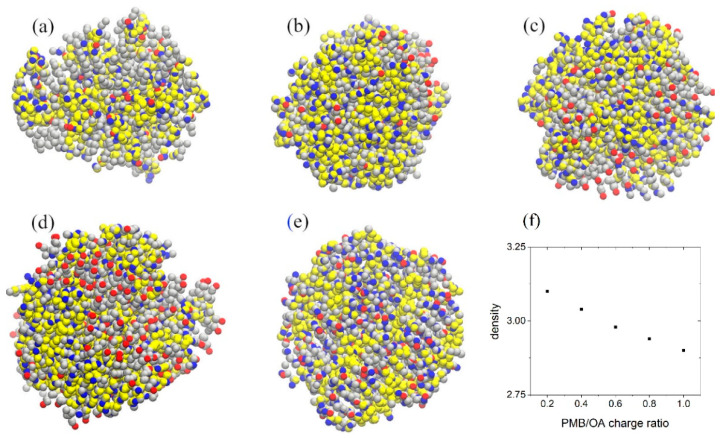
Snapshots of the simulation box, indicating ion paired clusters at PMB:OA charge ratios 1:0.2 (**a**), 1:0.4 (**b**), 1:0.6 (**c**), 1:0.8 (**d**), and 1:1 (**e**) in a system containing 200 PMB cations. Panel (**f**) indicates the number of water molecules in contact with PMB as a function of charge ratio.

An interesting feature of hydrophobic ion pairing is formation of a more hydrophobic complex than the drug (PMB). To investigate the influence of the concentration of hydrophobic counterion on the formation of hydrophobic ion paired clusters, we prepared samples consisting PMB and OA, in which the ratio of PMB:OA concentrations varied between 1:1 (charge ratio 1:0.2) to 1:5 (charge ratio 1:1). The well-formed clusters at different charge ratios are depicted in Figure 7. At lower PMB:OA charge ratios, the ion paired clusters are smaller and less compact. Increasing the PMB:OA charge ratio forms bigger and more compact clusters. Meanwhile, with increases in the charge ratio, more OA ions accumulate at the surface of the cluster. To calculate the degree of hydrophobicity of the complex, we counted the number of water molecules in contact (within a distance, $r_{C}$ from the cluster surface) with the PMB ions. The results in Figure 7f reveal that, with an increasing charge ratio, the number of water molecules in contact with PMB ions decreases. In other words, the fraction of the surface of clusters covered with hydrophobic OA ions increases with an increasing charge ratio. At a charge ratio 1:1, almost the maximum hydrophobicity is achieved; a further increase in the charge ratio does not increase the complex hydrophobicity. As expected, more hydrophobic complexes are formed in water–ethanol solvent than in pure water, i.e., the favorable hydrophobic–hydrophobic interactions between the OA tail and ethanol facilitate cluster surface coverage with the OA.

### 3.5. Cluster Stability and Drug Release Rate

**Figure 8:**
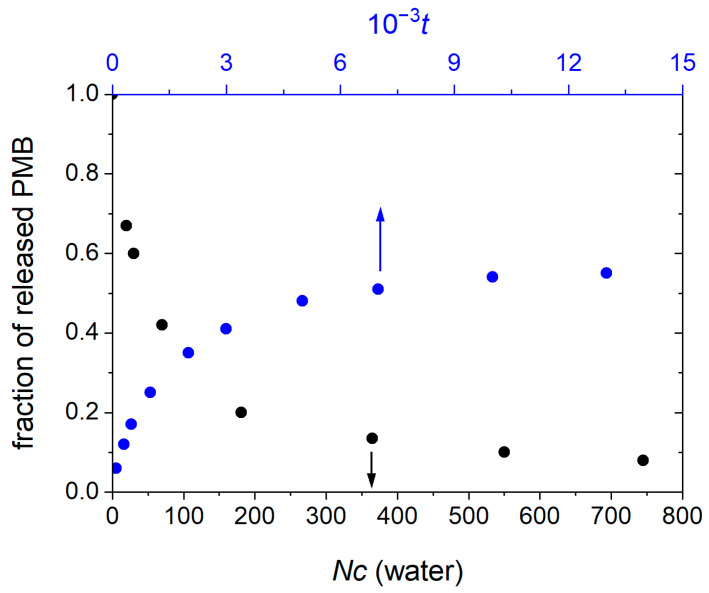
Dependence of the fraction of released PMB in the ion paired cluster in 40 mol% ethanol–water mixture on the cluster size in water, for clusters of various sizes (black points). Time-dependence of the fraction of PMB released from a hydrophobic ion paired cluster of size ≈ 200 PMB (the PMB:OA charge ratio is 1:1), immersed in a 165 mM aqueous NaCl solution (blue points).

Similar to the methods used in a previously published study [49], we investigated cluster stability by transferring an ion paired cluster into an ethanol–water mixture containing 40 mol% ethanol. The ion exchange between the ion paired cluster and ions in the solution causes the release of some PMB and OA to the solution. With the passage of time, more PBM and OA are exchanged with the solution and the cluster shrinks in size. Eventually a dynamic equilibrium establishes between the PMB and OA ions in the cluster and those in the solution. Figure 8 shows the fraction of released PMB from the ion paired cluster in 40 mol% ethanol vs. the initial size of the cluster (in pure water) for clusters of various sizes. The results show that smaller clusters more easily dissolve in ethanol than do the bigger ones.

Upon transferring the ion paired cluster to the aqueous ethanol solution, the alcohol causes cluster dissolution. At low ethanol concentrations, the alcohol accumulates around both the internal and external surface layers of the ion paired cluster. The surface layer acts as a secondary barrier against alcohol permeation into the ion paired cluster. At higher concentrations, ethanol further penetrates to the core of the cluster and causes it to swell. The core of the ion paired cluster acts as the primary barrier for ethanol penetration. Upon ethanol penetration, the cluster swells and, hence, further facilitates water penetration into it. In other words, the fraction of ethanol in the cluster increases disproportionately with increasing ethanol mole fraction in the surrounding solvent. This synergistic effect of water and ethanol for penetration into the ion paired cluster is in agreement with previous reports on the stabilities of pure and mixed [29] membranes of dipalmitoylphosphatidylcholine, dioleoylphosphatidylcholine, and dimyristoylphosphatidylcholine in the presence of ethanol.

Another important factor determining the dosing frequency in in vivo active concentrations and overall therapeutic effectiveness is the drug release rates from ion paired clusters. Similar to experimental results, the release rate of PMB was investigated by transferring the ion paired clusters into phosphate-buffered saline, modeled as a 165 mM sodium chloride solution, corresponding to the ionic strength of the serum. Figure 8 indicates the release rate of PMB from a hydrophobic ion paired cluster of size ≈ 200 PMB at the PMB:OA charge ratio 1:1. Upon transferring the ion paired cluster into the aqueous NaCl solution, an initial fast release followed by a regime of slow release rate is observed. The PMB cations at the surface of the cluster are exchanged with the hydrated Na^+^ in the solution. This step involves partial exchange of OA anions with the hydrated Cl^−^ as well. However, due to the exchange of PMB cation with the solution, the concentration of OA ions at the cluster surface increases [48], which acts as a barrier for the drug exchange rate, hence decreasing the release rate, in agreement with previously reported findings [48,50,51].

### 3.6. Kinetics of Ion Pair Formation

In equilibrium, the size of ion paired aggregates fluctuates around an average value. Recent experimental findings [52,53] and simulation reports on the formation of micellar aggregates [34,54] and colloid crystallization [55,56] found evidence for the variation of aggregate size through stepwise addition/removal of molecules to and from the cluster. To examine the validity of this mechanism for variation of ion paired clusters, the dynamics of PMB and/or OA exchange between a cluster of size *N* and the surrounding solution is investigated in terms of the following continuous, $S\left(t\right)$, and intermittent, $C\left(t\right)$, correlation functions:

(8) $$C\left(t\right)=\frac{\langle h\left(t\right)h\left(0\right)\rangle }{\langle h\left(0\right)h\left(0\right)\rangle }$$

and

(9) $$S\left(t\right)=\frac{\langle H\left(t\right)h\left(0\right)\rangle }{\langle h\left(0\right)h\left(0\right)\rangle }$$

where *t* is time, $h\left(t\right)$ is a binary function; $h\left(t\right)$ = 1 if a PMB ion is involved in an ion paired cluster at time *t* (irrespective of possible exchanges between the solution in the interim) and $h\left(t\right)$ = 0, otherwise. The binary function $H\left(t\right)$ = 1 if a particular PMB ion is continuously involved in the cluster during the time *t*, and $H\left(t\right)$ = 0, otherwise. On this basis, the continuous correlation function $S\left(t\right)$ measures the probability that a particular PMB/OA ion involved in a cluster of size *N* at time *t* = 0 continuously remains in the same cluster until time *t*. The intermittent correlation function, on the other hand, measures the probability that PMB/OA ions involved in a cluster of size *N* at time *t* = 0 are involved in the same cluster at time *t*. Obviously, the function $S\left(t\right)$ decays faster than the function $C\left(t\right)$. The relaxation time for the decay of function $S\left(t\right)$ is called the lifetime and that for the function $C\left(t\right)$ is called the structural relaxation time of a cluster.

**Figure 9:**
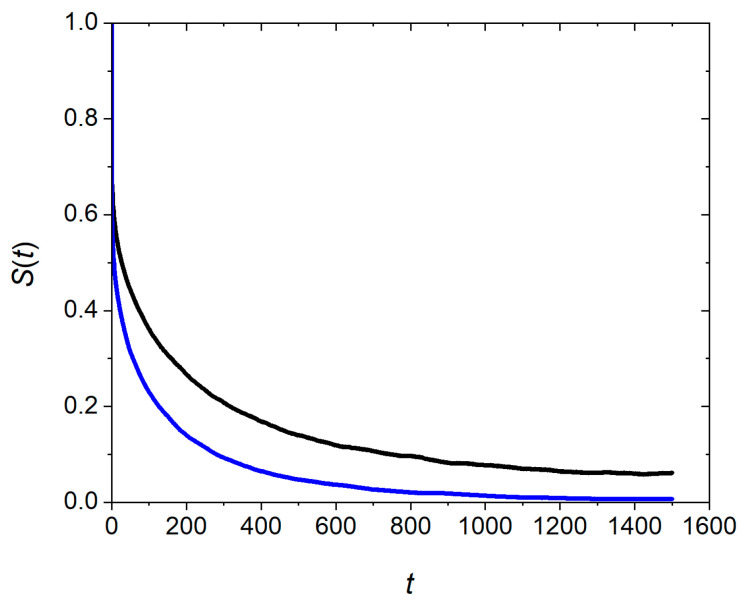
Decay of continuous correlation function, $S\left(t\right)$, for exchange of polymyxin B/oleate between the ion paired cluster and the solution. The cluster sizes are 758 (black curve) and 365 (blue curve).

**Figure 10:**
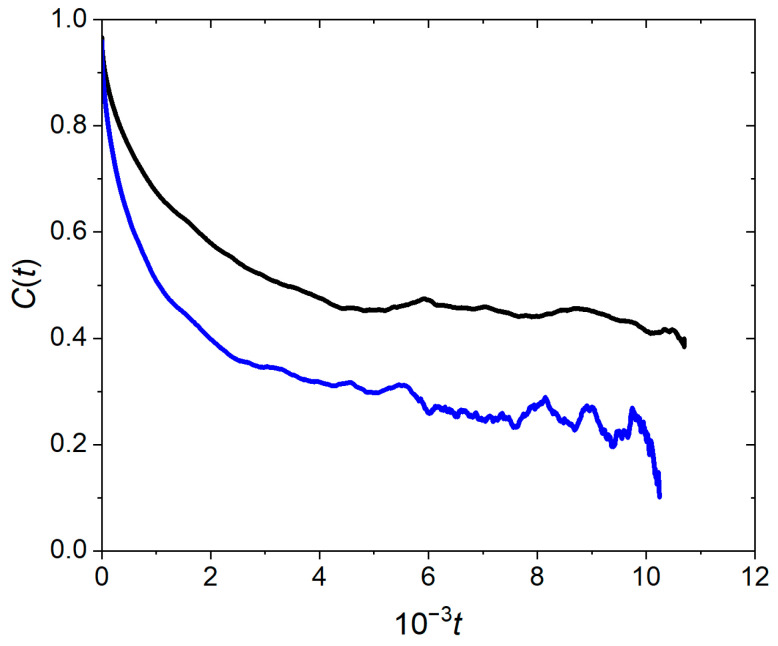
Decay of intermittent correlation function, $C\left(t\right)$, for exchange of polymyxin B/oleate between the ion paired cluster and the solution. The cluster sizes are 758 (black curve) and 365 (blue curve).

The correlation functions $S\left(t\right)$ and $C\left(t\right)$ for two cluster sizes are plotted in Figure 9 and Figure 10. The decay rate of the function $S\left(t\right)$ characterizes the rate of exchange of the PMB/OA ions on the surface of the cluster with those in the solution. The relaxation time, τ, depends on the cluster size; τ = 195 for *N* = 758 and τ= 78 for *N* = 365. The function $C\left(t\right)$ obeys an initial fast decay followed by a regime of slow decay rate. When PMB/OA ions exchange between a cluster and the surrounding solution, diffusion brings some of the ions back to the cluster surface. Therefore, the slow decay rate regime is connected to diffusion [17]. The structural relaxation time, $\tau_{R}$, for function $C\left(t\right)$ varies from 5000 for clusters of size 365 to 2.8 × 10^4^ for clusters of size 758. Compared to the lifetimes for clusters, the structural relaxation times are much longer and are more sensitive to the cluster size. The marked difference between the structural relaxation time and the lifetime of clusters is indicative of distinct motion regimes of PMB/OA ion exchange between the clusters and the solution. The lifetime of a cluster refers to dissociation/association of individual PMB/OA ions from/to the cluster. The structural relaxation time of a cluster, however, refers to establishment of equilibrium between the PMB/OA ions in the solution and those involved in the cluster through diffusion of PMB/OA ions in the solution.

It is worth mentioning that the times quoted in the DPD simulations are only qualitative measures of dynamics of similar processes. In all coarse-grained schemes, due to the removal of atomistic details of the system, the dynamics are artificially accelerated [57,58]. Therefore, the relaxation times reported here are shorter than the corresponding experimental relaxation times. To be able to provide an estimate of realistic relaxation times (comparable with experimental findings), we can calculate the degree of dynamic acceleration in our DPD simulations. For this purpose, we compare the calculated diffusion coefficient of water with that of experimental findings. The calculated diffusion coefficient of water beads (each bead consisting of four water molecules) is 1.64 × 10^−3^ cm^2^/s and the experimental diffusion coefficient of water is 2.43 × 10^−5^ cm^2^/s [59]. The scaling factor for conversion of the DPD time scale to the actual time scale corresponds to 4 × 1.64 × 10^−3^ (cm^2^/s)/2.43 × 10^−5^ (cm^2^/s) ≈ 270, where the factor 4 represents that 4 water molecules are mapped into a single water DPD bead. Based on this time scaling constant, the lifetimes for clusters of sizes 365 and 758 are ≈80 ns and ≈200 ns, respectively. Similarly, the structural relaxation times of clusters for clusters of sizes 365 and 758 correspond to ≈5 μs and ≈30 μs, respectively.

For clusters formed in the mixed ethanol–water mixtures, both functions, $S\left(t\right)$ and $C\left(t\right),$ decay faster than those in the pure water. The slow relaxation regime, which pertains to the establishment of equilibrium between the PMB/OA ions in the solution and those involved in the cluster, is connected to the diffusion of PMB/OA ions from the solution to the cluster surface and vice versa. The time scale for this process (μs scale) provides useful information on the rate of drug release from the hydrophobic ion paired complex. The composition of the interface (water and/or water–ethanol mixtures) between the drug aggregates and the surrounding solvent is a determining factor for the release rate [60].

## 4. Conclusions

A dissipative particle dynamics (DPD) model, based on the four-to-one Martini mapping scheme [27,28], was constructed to study hydrophobic ion pairing in polymyxin B (PMB) and oleate (OA) ions. Solvent (water and ethanol), and hydrated counterions were explicitly included in the simulations. Moreover, a Gaussian charge distribution method for inclusion of electrostatic interactions in DPD [34] was employed to calculate Coulombic interactions between the charged DPD beads.

Our findings are indicative of a step-growth mechanism of cluster growth in the PMB/OA system. In the initial stages of assembly, the oppositely charged PMB and OA ions attract each other to form initial (unstable) precursors, which further convert to more stable clusters. In this regime, the cluster size varies smoothly with time. In the subsequent step, smaller clusters collide to form bigger clusters. In this stage, the clusters do not grow unless diffusion brings them together. This step-growth mechanism of cluster growth is similar to that for nucleation/crystallization of colloidal particles [42,55,56].

The ratio of PMB:OA concentrations (the charge ratio) is an important factor, which controls the hydrophobicity of the cluster. At lower charge ratios, the ion paired clusters are smaller and less compact. Increasing the charge ratio causes formation of bigger and more compact clusters, in which more OA ions accumulate at the surface of the cluster. The maximum hydrophobicity is achieved at a PMB:OA charge ratio of 1:1. The tendency for formation of a hydrophobic ion paired complex also depends on the solvent philicity. The clustering efficiency in mixed water–ethanol solvents decreases with the increasing ethanol content of water.

We observed two distinct relaxation processes for the dynamics of PMB/OA ion exchange between the hydrophobic cluster and the surrounding solution. The faster relaxation process corresponds with the exchange of PMB/OA ions at the surface of the cluster with the surrounding. The slower relaxation process, on the other hand, pertains to the establishment of equilibrium between the PMB/OA ions in the solution and those involved in the cluster, and is connected to the diffusion of PMB/OA ions from the solution to the cluster surface and vice versa. The time scale for this process provides useful information on the rate of drug release from the hydrophobic ion paired complex.

## Figures and Tables

**Table 1:** DPD interaction parameters. (a) nonbonded interactions parameter, $A_{ij}$*.

Bead Type	CH_2_CH_2_NH_3_^+^	CH_2_COO^−^	NHCOCH	CH_3_CHOH	C_4_H_8_	(H_2_O)_4_
CH_2_CH_2_NH_3_^+^	100	100	100	102.5	125.5	100
CH_2_COO^−^	100	100	100	102.5	125.5	100
NHCOCH	100	100	100	102.5	110	100
CH_3_CHOH	102.5	102.5	102.5	100	105	102.5
C_4_H_8_	125.5	125.5	110	105	100	125.5
(H_2_O)_4_	100	100	100	102.5	125.5	100

* The interaction parameters for Na^+^(H_2_O)_4_, Cl^−^(H_2_O)_3_, and ethanol (C_2_H_5_OH)_1.24_ beads are the same as those for CH_2_CH_2_NH_3_^+^, CH_2_COO^−^, and CH_3_CHOH, respectively.

**Table 2:** Description of systems simulated in this work.

PMB^5+^	OA^−^	Cl^−^	Na^+^	Water	Total	Length of Cubic Box
800	4000	4000	4000	1,192,000	1,238,400	74.26
800	3200	4000	3200	1,196,800		
800	2400	4000	2400	1,201,600		
800	1600	4000	1600	1,206,400		
800	800	4000	800	1,211,200		
600	3000	3000	3000	1,119,400	1,228,800	74.12
600	2400	3000	2400	1,197,600		
600	1800	3000	2800	1,201,200		
600	1200	3000	1200	1,204,800		
600	600	3000	600	1,208,400		
400	2000	2000	2000	1,196,000	1,219,200	73.84
400	1600	2000	1600	1,198,400		
400	1200	2000	1200	1,200,800		
400	800	2000	800	1,203,200		
400	400	2000	400	1,205,600		
200	1000	1000	1000	1,198,000	1,209,600	73.70
200	800	1000	800	1,199,200		
200	600	1000	600	1,200,400		
200	400	1000	400	1,201,600		
200	200	1000	200	1,202,800		
80	400	400	400	1,199,200	1,203,840	73.55
80	320	400	320	1,199,680		
80	240	400	240	1,200,160		
80	160	400	160	1,200,640		
80	80	400	80	1,201,120		
40	200	200	200	1,199,600	1,201,920	73.55
40	160	200	160	1,199,840		
40	120	200	120	1,200,080		
40	80	200	80	1,200,320		
40	40	200	40	1,200,560		
30	150	150	150	1,199,700	1,201,440	73.70
30	120	150	120	1,199,880		
30	90	150	90	1,200,060		
30	60	150	60	1,200,240		
30	30	150	30	1,200,420		
20	100	100	100	1,199,800	1,200,960	73.69
20	80	100	80	1,199,920		
20	60	100	60	1,200,040		
20	40	100	40	1,200,160		
20	20	100	20	1,200,280		
10	50	50	50	1,199,920	1,200,480	73.68
10	40	50	40	1,199,960		
10	30	50	30	1,200,020		
10	20	50	20	1,200,080		
10	10	50	10	1,200,140		

## Data Availability

All data and associated protocols are included in the manuscript.
